# Design of a lightweight universal talus implant using topology optimization

**DOI:** 10.3389/fbioe.2023.1228809

**Published:** 2023-08-24

**Authors:** Ahmed H. Hafez, Marwan El-Rich, Tao Liu, Nadr Jomha, Andreas Schiffer

**Affiliations:** ^1^ Department of Mechanical Engineering, Khalifa University, Abu Dhabi, United Arab Emirates; ^2^ Healthcare Engineering Innovation Center (HEIC), Khalifa University of Science and Technology, Abu Dhabi, United Arab Emirates; ^3^ Human Performance Lab, Faculty of Kinesiology, University of Calgary, Calgary, AB, Canada; ^4^ Faculty of Medicine and Dentistry, University of Alberta, Edmonton, AB, Canada

**Keywords:** talus implant design, bioinspired design, total talus replacement, finite element analysis, topology optimization, contact pressure, cartilage

## Abstract

Total talus replacement is a promising alternative treatment for talus fractures complicated by avascular necrosis and collapse. This surgical option replaces the human talus bone with a customized talus implant and can maintain ankle joint functionality compared to traditional treatment (e.g., ankle fusion). However, the customized implant is costly and time-consuming due to its customized nature. To circumvent these drawbacks, universal talus implants were proposed. While they showed clinically satisfactory results, existing talus implants are heavier than biological talus bones as they are solid inside. This can lead to unequal weight between the implant and biological talus bone, and therefore leading to other complications. The reduction of the implants’ weight without compromising its performance and congruency with surrounding bones is a potential solution. Therefore, this study aims to design a lightweight universal talus implant using topology optimization. This is done through establishing the loading and boundary conditions for three common foot postures: neutral, dorsi- and plantar-flexion. The optimized implant performance in terms of mass, contact characteristics with surrounding joint cartilage and stress distributions is studied using a 3D Finite Element (FE) model of the ankle joint. The mass of the optimized implant is reduced by approximately 66.6% and its maximum stresses do not exceed 70 MPa, resulting in a safety factor of 15.7. Moreover, the optimized and solid implants show similar contact characteristics. Both implants produced peak contact pressures that were approximately 19.0%–196% higher than those produced by the biological talus. While further mechanical testing under *in-vivo* loading conditions is required to determine clinical feasibility, preliminarily, the use of a lightweight universal implant is expected to provide the patient with a more natural feel, and a reduced waiting period until surgery.

## 1 Introduction

The talus bone, with its unique geometry and large articular surface, plays a significant role in load transmission and foot movement as it serves as the connection point between the leg and the foot (Liu et al., 2020). Given its poor blood supply and large cartilage-covered surface area, coupled with the non-existence of muscular or tendinous attachments, the talus is more susceptible to avascular necrosis (AVN). AVN is the death of bone tissue due to restricted blood supply (Tonogai et al., 2017), and may be a result of fractures (Trovato et al., 2018), high-energy injuries (Katsui et al., 2019), trauma, steroid use, metabolic or idiopathic causes (Bowes et al., 2019), and osteosarcoma (bone cancer) (Huang et al., 2021). Ultimately, talar collapse manifests in the form of ankle joint incongruity leading to pain, stiffness and restricted movement (Bowes et al., 2019).

A common surgical treatment is ankle arthrodesis (fusion) where the talus is fixated to the tibia or to both the tibia and calcaneus. While this procedure provides acceptable pain relief, it results in the loss of hindfoot and ankle movement as well as increased stresses on the surrounding joints (Bowes et al., 2019). A more favorable alternative is total talus replacement (TTR) surgery, which results in a higher rate of pain relief (Tonogai et al., 2017), preserved range of motion and joint function as well relatively easier surgeries and reduced recovery periods (Hussain, 2020). Since TTRs are typically patient-specific, the custom-made implant’s design process can be time-consuming and costly, resulting in increased periods between surgical decision and implantation (Liu et al., 2020). To solve the drawbacks, universal talar prostheses have been previously developed and were proved to be feasible (Trovato et al., 2018; Bowes et al., 2019; Trovato, 2016; Trovato et al., 2017; Liu et al., 2022a). Due to the universal nature, these types of implants can be mass produced. Both the waiting time between diagnosis and surgery as well as the associated design and production costs can be reduced (Trovato et al., 2017). However, when comparing the weight of the talus implant to the biological talus bone, existing universal talus implants are generally up to several times heavier, given the use of ceramics and metals as implant materials (West and Rush, 2021). This can lead to unequal weight of the left and right foot and potential complications.

In order to enhance implant structures, topology optimization (TO) is typically used. TO is a procedure that optimizes material distribution in a defined design space in order to achieve higher performance structures, typically ones with lighter weight while maintaining mechanical properties (Kladovasilakis et al., 2020). For TTRs, a single study is known to have employed TO in their design process. In that study, a comparison was made between a topologically optimized scaffold and a rational scaffold of the inner structure of a talus replacement in three postures corresponding to peak gait cycle loads. The implant used was a recreation of a cadaveric talus, and the simulation excluded the fibula as well as the adjacent bones’ cartilages (Kang et al., 2022). This exclusion likely decreases the accuracy of the resulting stress distributions, thereby affecting the optimized implant geometry and expected performance under more anatomically-accurate conditions.

This study focused on the design of a universal talus implant, under three loading scenarios, using topology optimization to obtain an enhanced structure that benefits from the advantages of a universal implant as well as addresses some limitations of the aforementioned study by including the fibula and the bones’ cartilages in the model. The optimized implant’s performance (mass, stresses and contact pressures) is then assessed, in comparison with the non-optimized solid implant and biological talus, using finite element analysis (FEA).

## 2 Materials and methods

### 2.1 Geometry acquisition

The universal talus implant’s geometry was obtained from an earlier study (Trovato et al., 2017) where a talus (among 91 tali) with the least total deviation from the rest of the tali was selected, and was uniformly further scaled up by 0.5 mm to compensate for the cartilage existing on a biological talus bone.

The ankle joint geometry, including the biological talus bone, was obtained from an earlier study (Trovato et al., 2018) where a dissected cadaveric foot (right side) was CT scanned under three postures: +20° dorsiflexion (DF, foot pointed upwards), 0° neutral standing (NS, standing position), and −20° plantarflexion (PF, foot pointed downwards). The angles were selected to represent a wide range of flexion angles of the foot where the postures are typically experienced when ascending/descending stairs, for example, (Brockett and Chapman, 2016). The obtained images were then imported into MIMICS (Materialize, NV, Belgium, Version 20.0) where the bones were created, and then cleaned using Geomagic (3D Systems^®^, Morrisville, USA, Version 2014) to ultimately obtain the 3D geometry. The cartilage, presented in greater detail in Section 2.3, was created using Hypermesh (Altair^®^, Troy, United States, Version 2021), by extruding the shell elements on the articular surfaces of the adjacent bones, as well as the biological talus, by 1.5 mm (Liu et al., 2022a). Throughout the study, the term ‘ankle joint’ is used to represent all the five bones (including the navicular and calcaneus), not strictly the anatomical ankle joint composed of the talus, tibia, and fibula. Additionally, the term ‘adjacent bones’ refers to the aforementioned ankle joint without the talus. Finally, unless stated otherwise, the terms ‘implant’ and ‘universal implant’ are used interchangeably.

### 2.2 Biological talus bone and universal implant setup

**FIGURE 1:**
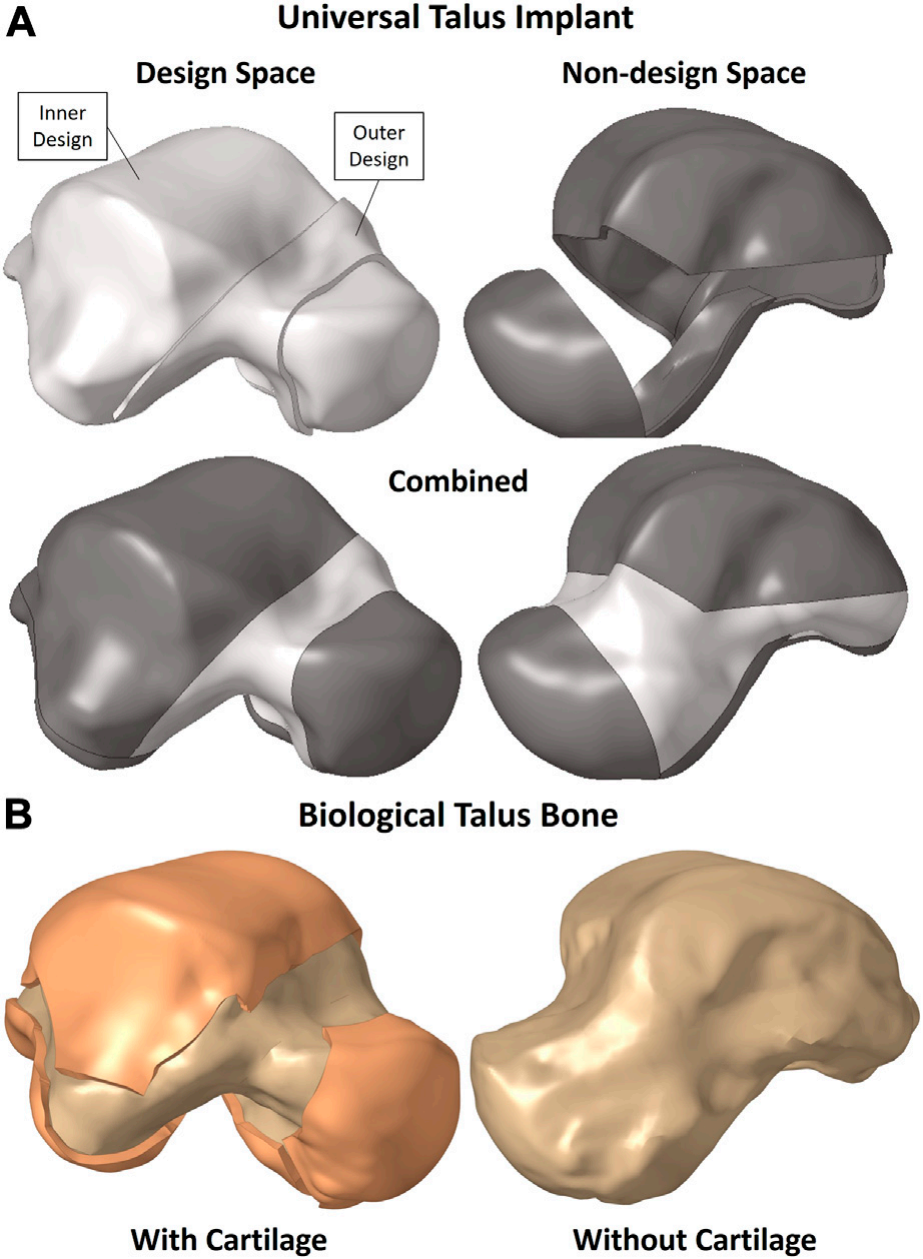
**(A)** Universal talus implant and **(B)** biological talus bone setup.

The solid implant geometry, shown in Figure 1A, was partitioned to easily allow its modification for different FEA setups as well as the optimization while preserving the mesh for consistency. It was divided into the design space (in white) and non-design space (in grey). The outer sections, namely, the outer design space and entire non-design space, are 1 mm-thick solids. The biological talus bone, presented in greater detail in Section 2.3, is shown in Figure 1B with and without its cartilage. For the purposes of this study, the biological talus was used strictly for comparison, not optimization.

The partitions were not randomly created; rather they were defined to maintain the implant’s function in terms of contact between the non-design space partitions with their respective adjacent bones’ cartilages while accounting for additive manufacturing (AM) constraints. The main consideration was to allow the metal powders used in powder-based AM to be removed, hence the availability of an outer design space as well instead of only having an enclosed inner design space where the powders would remain trapped.

### 2.3 Finite element model

The following section describes the setup for both implants, namely, the optimized and solid implants, in addition to the biological talus.

#### 2.3.1 Solid implant and ankle joint FE setup

**FIGURE 2:**
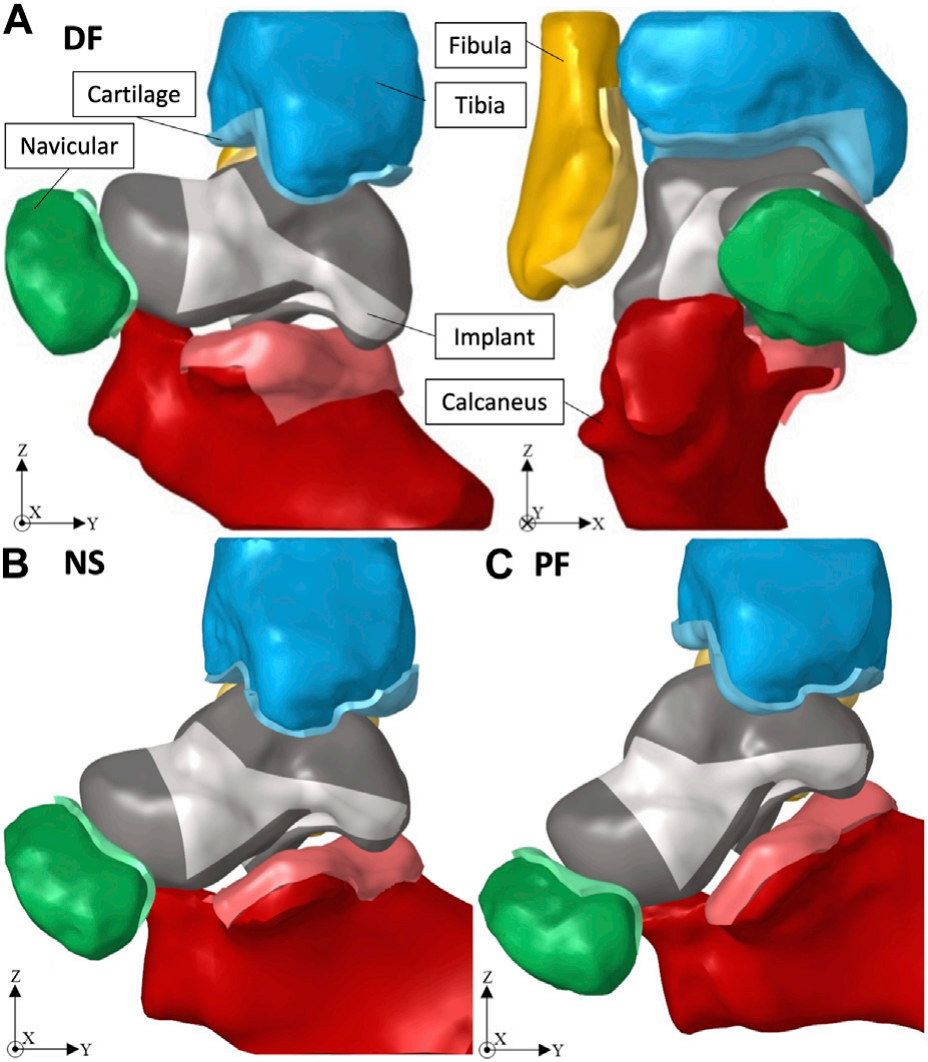
Ankle joint in **(A)** +20° DF, **(B)** 0° NS, and **(C)** −20° PF.

Given that the obtained ankle joint’s 3D geometry was of the biological bones, the solid implant was ‘best-fit’ aligned with the biological talus using Geomagic, and then the adjacent bones were translated away from the implant to avoid interference when creating their cartilage layers. Figure 2 shows the ankle joint FE setup, with the solid implant, in dorsiflexion (DF, +20°), neutral standing (NS, 0°), and plantarflexion (PF, −20°) respectively.

The simulation of each posture, using Optistruct solver (Altair^®^, Troy, United States, Version 2021), includes three consecutive, large-displacement, non-linear static load steps: 1) the adjacent bones are returned to their respective CT scan position to establish contact with the implant which is fixed in place; 2) the implant freely adjusts itself with the adjacent bones which remain fixed in their positions at the end of load step 1; 3) a compressive force of 2000 N is applied to the tibia in the direction of gravity (negative *Z*-axis). Note that the magnitude of the compressive force (2000 N) equates to approximately three to four times a person’s weight and was chosen in accordance with a previous study (Liu et al., 2022a). More complex or extreme loading conditions were not considered in this work. Both the tibia and fibula are equation-constrained to move together in the *Z*-axis to mimic their realistic combined motion (Liu et al., 2022a).

All the defined loads, displacements and equation constraints are applied to reference points representing rigid body motion of a set of elements on the adjacent bones. The only exception is the implant whose defined displacements are applied to the nodes on the outer surface of its outer design space.

To reduce computational difficulties, the non-articular surfaces of the adjacent bones were meshed using three-node triangular shell elements while the articular surfaces were meshed using four-node quad elements. All the shell elements were assigned a thickness of 1 mm. For the cartilage, the articular surface’s four-node elements were extruded 1.5 mm, distributed over four equal layers, to create solid eight-node hexahedral elements.

Since the implant is an artificial replacement for the biological talus, no cartilage was created on its surface, rather its volume was uniformly scaled up by 0.5 mm to account for the natural cartilage thickness. For the non-design sections, their outer surfaces were first shell-meshed using four-node quad elements (for the main areas that will be in contact with the adjacent bones’ cartilages) while surrounded by three-node triangular elements. The shell mesh of the five aforementioned sections was then mapped across each respective solid section, from its outer to its inner surface, creating eight-node hexahedral and six-node pentahedral solid elements. For the outer-design section, since it does not establish any contact, its outer surface was meshed with quad-dominated mixed shell elements. Finally, the inner-design section was meshed using four-node tetrahedral solid elements.

Herein, the implant, cartilages, and bones were assumed to be homogeneous isotropic solids, and their assigned material models and properties are summarized in Table 1. Additionally, surface-to-surface contacts were defined between the cartilages of the adjacent bones and their related contact areas on the outer surfaces of the respective non-design space of the implants. A static friction coefficient, µ_s_ = 0.01 (frictionless contact), was defined between the contact pairs, and separation was allowed after contact (Liu et al., 2022a).

**TABLE 1:** Model material properties.

Component	Material Behavior	Mechanical Properties	
Bones (cortical bone)	Linear elastic	E = 19 GPa, ν = 0.3	Liu et al. (2022a)
Cartilage	Hyperelastic (Ogden)	µ_1_ = 2.43 MPa, *α*_1_ = 12.45, D_1_ = 0.176 $\frac{1}{MPa}$	Liu et al. (2022a)
Implant (Ti-6Al-4V)	Linear elastic	E = 107 GPa, ν = 0.323, ρ = 4,405 $\frac{kg}{m^{3}}$	Ansys Workbench (2020)

#### 2.3.2 Biological talus FE setup

As for the ankle joint FE setup involving the biological talus, a visual demonstration is shown in an earlier study (Liu et al., 2022a). A setup similar, in terms of loading and material properties, to that in Section 2.3.1 was utilized where the biological talus was in place of the solid implant. The selected rigid body set of elements were on the non-contact shell elements. The talus and its cartilage were meshed identically to the aforementioned adjacent bones (see Section 2.3.1). In this particular case, the surface contacts were defined between the adjacent bone cartilages and their related contact areas on the talus’ cartilage surface.

#### 2.3.3 Optimized implant FE setup

The optimized universal implant, presented further in Section 3.1, is visualized in isolation from the ankle joint in this study, as shown in Figure 5B. After TO (defined in Section 2.5), the updated finite element (FE) model of the ankle joint with the optimized implant, a setup identical to that of the solid implant in Section 2.3.1, was used for all three postures. The non-optimized (solid) implant was substituted with the optimized one in order to evaluate the performance of the latter. The only difference is the optimized implant’s mesh, which due to its complex geometry, was fully meshed using four-node tetrahedral solid elements.

### 2.4 Mesh sensitivity analysis

To verify that the simulation results are independent of the solid implant’s mesh, a sensitivity analysis was conducted. The ankle joint, in NS only, was simulated by varying the total number of elements of the implant while maintaining the mesh setup described in Section 2.3.1.

Based on the results, beyond 376,156 elements (100,083 nodes) the stress values varied minimally, where for at least a 4.2% increase in the total number of elements leads to an increase of at most 1.7% in either von Mises or contact stress, the solid implant was deemed to be mesh insensitive. Therefore, the model selected for further FE simulations and optimizations was the one with 414,111 elements (118,218 nodes). It possesses a sufficiently high number of elements, which will be required for a more detailed representation of the optimized topology; while it simultaneously has less elements, for computational efficiency, than the model with the finest mesh.

### 2.5 Topology optimization setup

The mesh and the material properties used for the implant are as described in Section 2.3.1. To further expand on Section 2.2, in the context of TO, the definitions of the design and non-design spaces, shown in Figure 1A, are important. The non-design space is the section that remains unchanged and is not optimized while the design space is the section that changes and is optimized, hence the partitions created in the implant.

**FIGURE 3:**
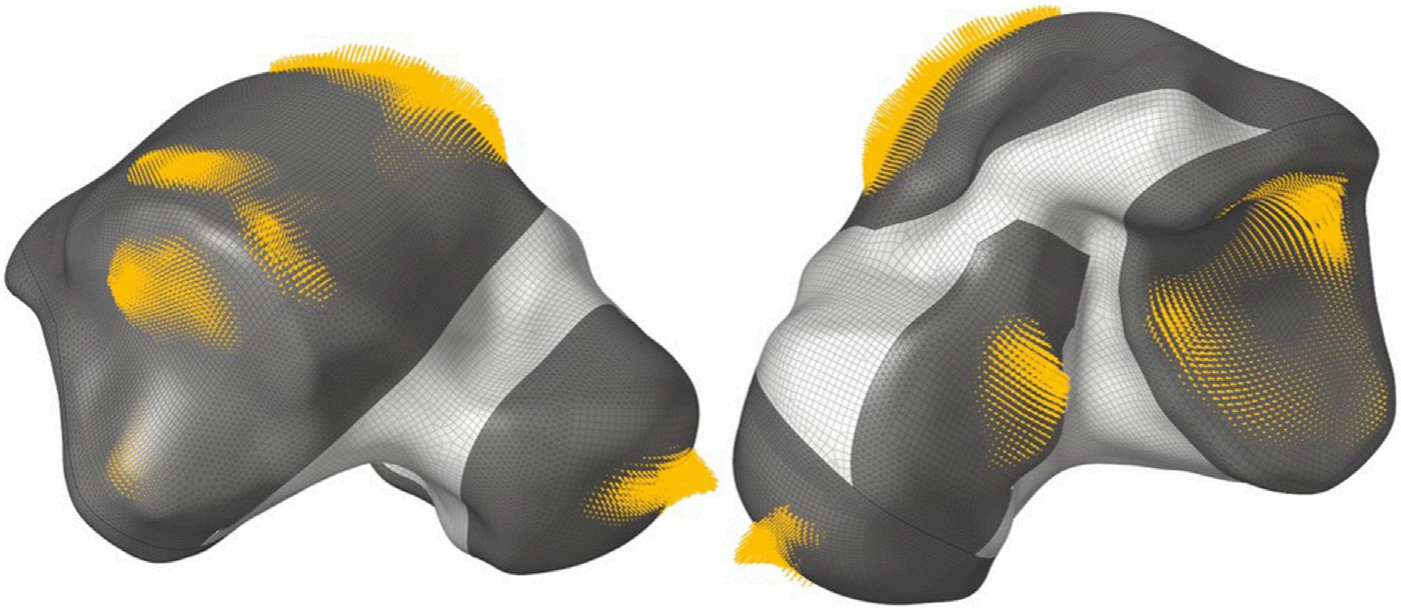
Nodal contact forces in dorsiflexion.

For the loading conditions of the implant, the output nodal contact forces on the non-design sections from the FEA of the implant (resulting from the analysis in Section 2.3.1), in all three postures, were directly applied as loads on the same nodes of the implant (since the same mesh was used). All nodal contact forces belonging to each posture were placed in a load step of their own for a total of three linear static load steps. For example, Figure 3 shows the DF load step, where the size of each arrow is proportional to the magnitude of each nodal contact force.

The inertia relief ‘INREL’ parameter was activated which allows the software to run the static analysis without constraints, and instead, the applied loads are balanced out by nodal accelerations (automatically determined by Optistruct).

For the TO setup, the objective was to minimize the total volume *V(* ***ρ*** *)* of the design space, which is the combination of both the ‘Inner Design’ and ‘Outer Design’ sections, as shown in Figure 1A. A von Mises (VM) static stress constraint was applied to the entire implant where the maximum VM stress of element *e*, $\sigma_{vm,e}$ , may not exceed 75 MPa. The TO problem can be formulated as follows:

$$minimize\;V\left(\rho \right)=\sum_{e=1}^{N}\rho_{e}\;w.r.t.\;\rho_{e}$$ (1)

$$subject\;to\;\sigma_{vm,e}\leq 75\;MPa$$ (2)

where

$$0.01\leq \rho_{e}\leq 1,where\;e=1,\ldots ,N$$ (3)

The design variable is the relative density *ρ* of each element in the design space, where the relative density is the ratio of the optimized element’s volume to the same element’s non-optimized volume (which is a solid). Each element *e*, ranging from 1 to *N* number of finite elements, is assigned the design variable *ρ*_*e*_.

The 75 MPa limit was selected to maintain a sufficiently high safety factor (SF, where SF is the ratio of the material’s yield strength, 1,100 MPa for Ti-6Al-4V (Ansys Workbench, 2020), to the maximum VM stress in the model) against yielding while still obtaining a relatively lightweight structure.

## 3 Results

### 3.1 Optimized geometry

**FIGURE 4:**
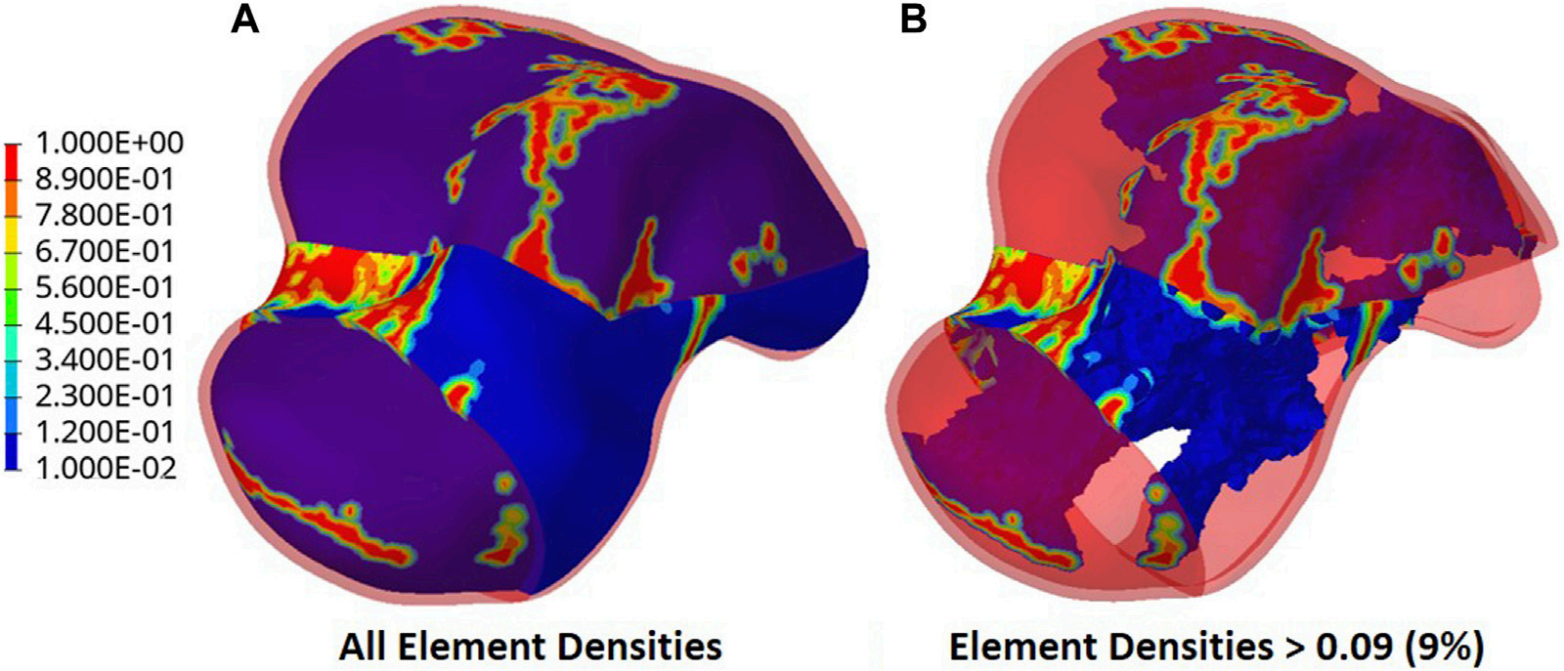
Optimized implant element density contours for **(A)** all densities and **(B)** densities >0.09.

The output of the optimization is the element density contour plot where the element densities (relative densities) of each element are shown. The relative density is the ratio of the optimized element’s volume to the volume of the same non-optimized element. The values range from 0.01 (1%) to 1 (100%), where a (near) zero value represents a void (no material) and a value of one represents a solid (material). The element density contour plot of the optimized implant is shown for all densities in Figure 4A, and densities >0.09 in Figure 4B. Since the non-design space is not optimized, it is made transparent in Figure 4 for better visualization.

**FIGURE 5:**
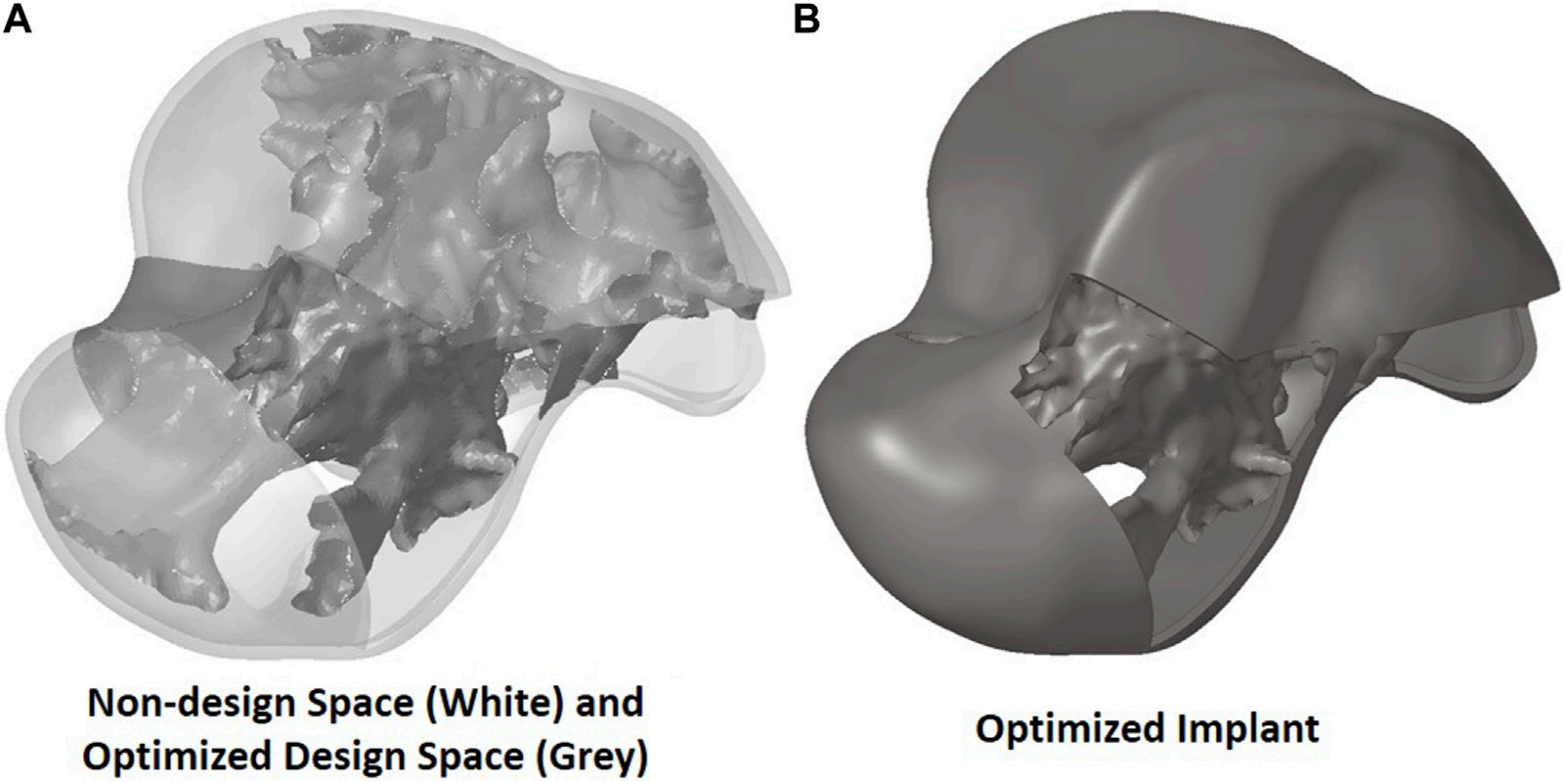
Optimized implant geometry **(A)** with non-design and optimized design spaces and **(B)** overall.

**FIGURE 6:**
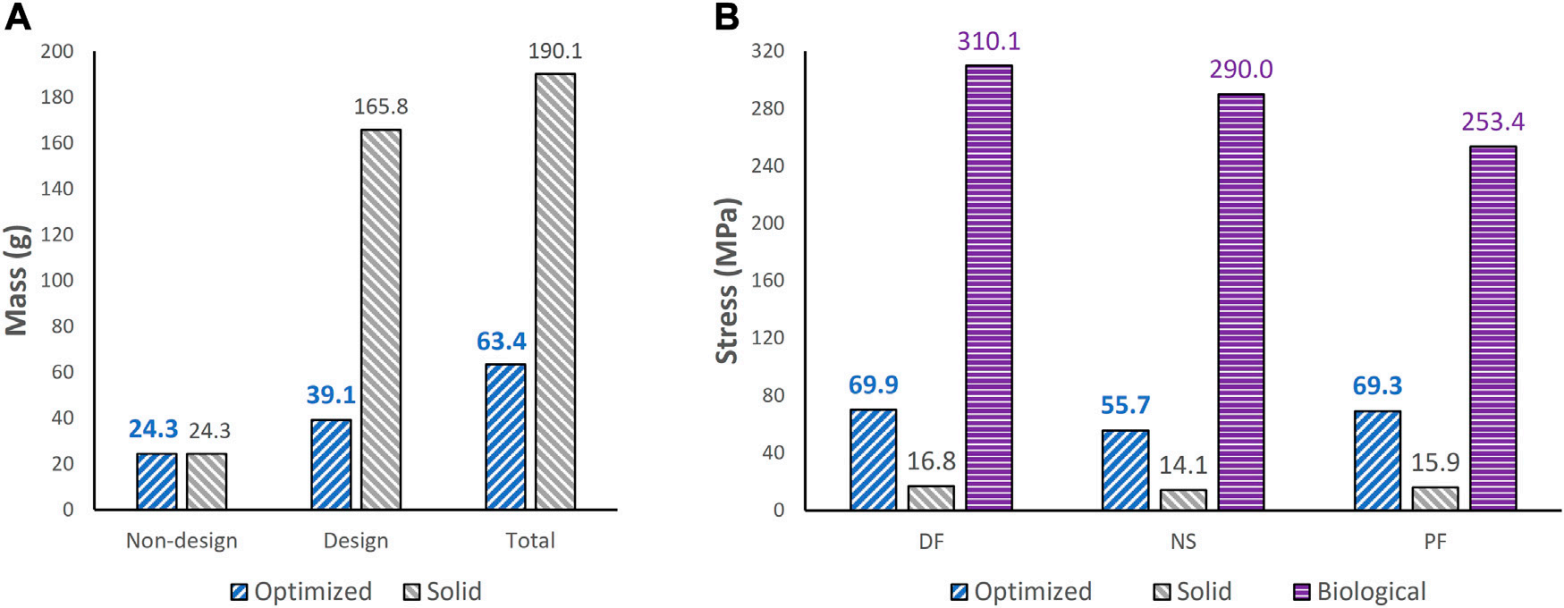
Comparisons of **(A)** mass between optimized and non-optimized (solid) implants and **(B)** maximum von Mises stress between optimized and solid implants, and biological talus.

To extract the surface of the optimized geometry, the Hypermesh post-processing tool ‘OSSmooth’ was used and an iso-density boundary surface was extracted with a selected threshold density of 9%. The selected threshold density was based on requiring the removal of low-density solid elements while maintaining connections in some areas between the design and non-design space. The resulting optimized geometry’s non-design and optimized design spaces are shown in Figure 5A while the solidified geometry, used for further analysis as defined in Section 2.3.3, is shown in Figure 5B. The masses of the design and non-design spaces, as well as the total masses of both the optimized and solid (non-optimized) implants are plotted in Figure 6A for a material density of 4,405 $\frac{kg}{m^{3}}$ , corresponding to Ti-6Al-4V (Ansys Workbench, 2020).

### 3.2 Stress comparisons

**FIGURE 7:**
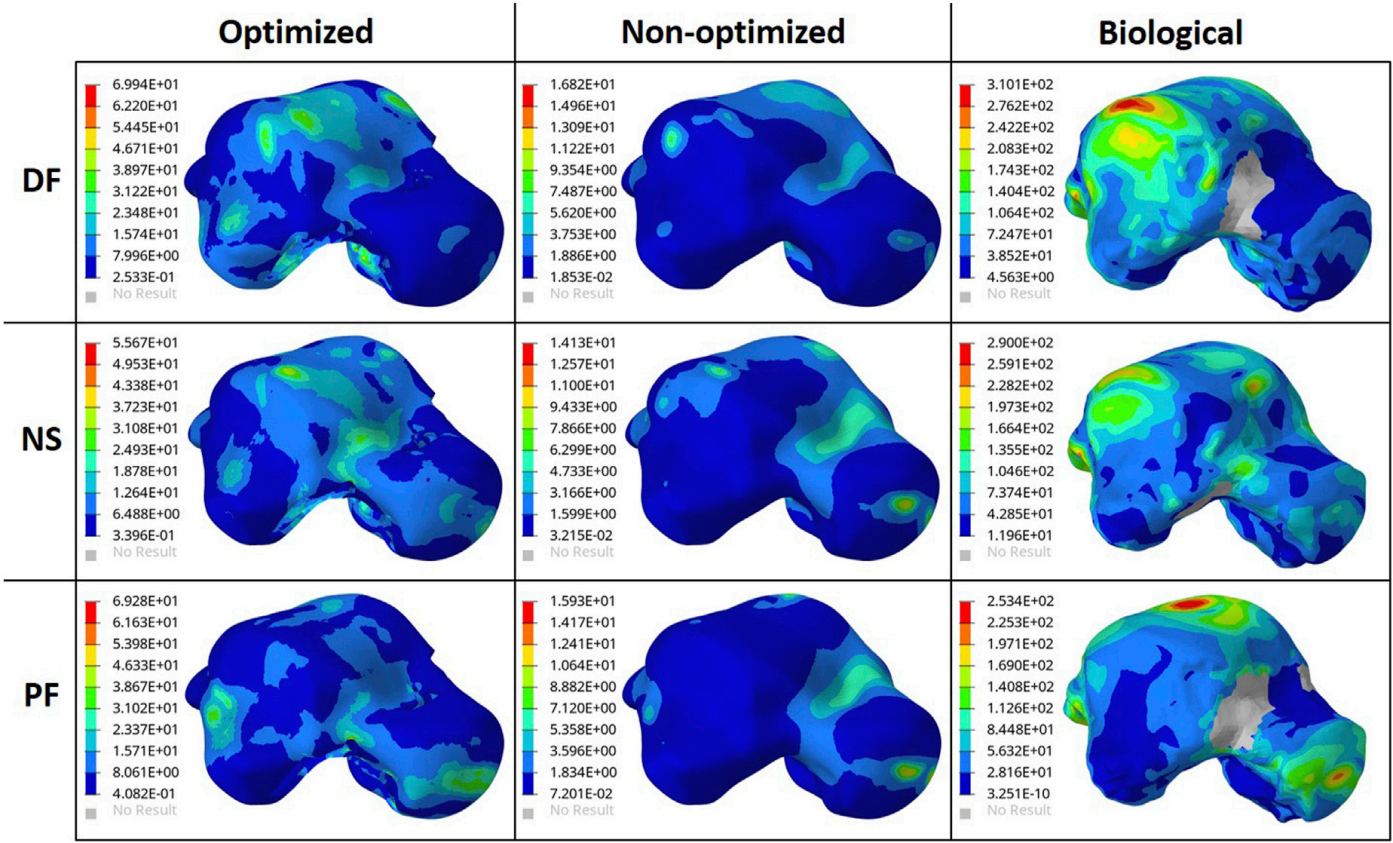
Von Mises Stress (MPa) contours for the optimized and non-optimized (solid) implants, and biological talus.

To ensure the safety of the optimized implant, in addition to verifying that the optimization stress constraints were satisfied, a VM stress evaluation was conducted. For comparison of the optimized and solid implants as well as the biological talus, the maximum VM stresses are plotted in Figure 6B and the stress contours are shown in Figure 7 for all three postures. Note that the cartilage of the biological talus is hidden in Figure 7 for visualization and ease of comparison, and the grey sections were defined as rigid body sets, hence the nonexistence of stress contours.

### 3.3 Contact pressure comparisons

**FIGURE 8:**
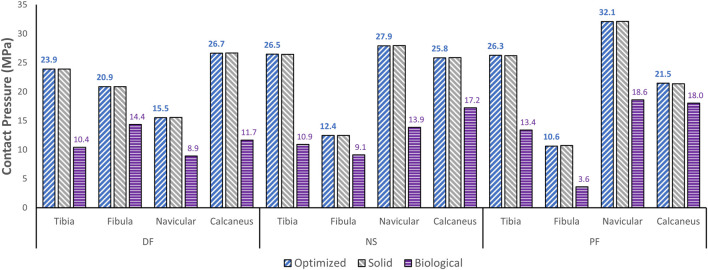
Maximum contact pressure comparisons between the optimized and solid implants, and biological talus.

**FIGURE 9:**
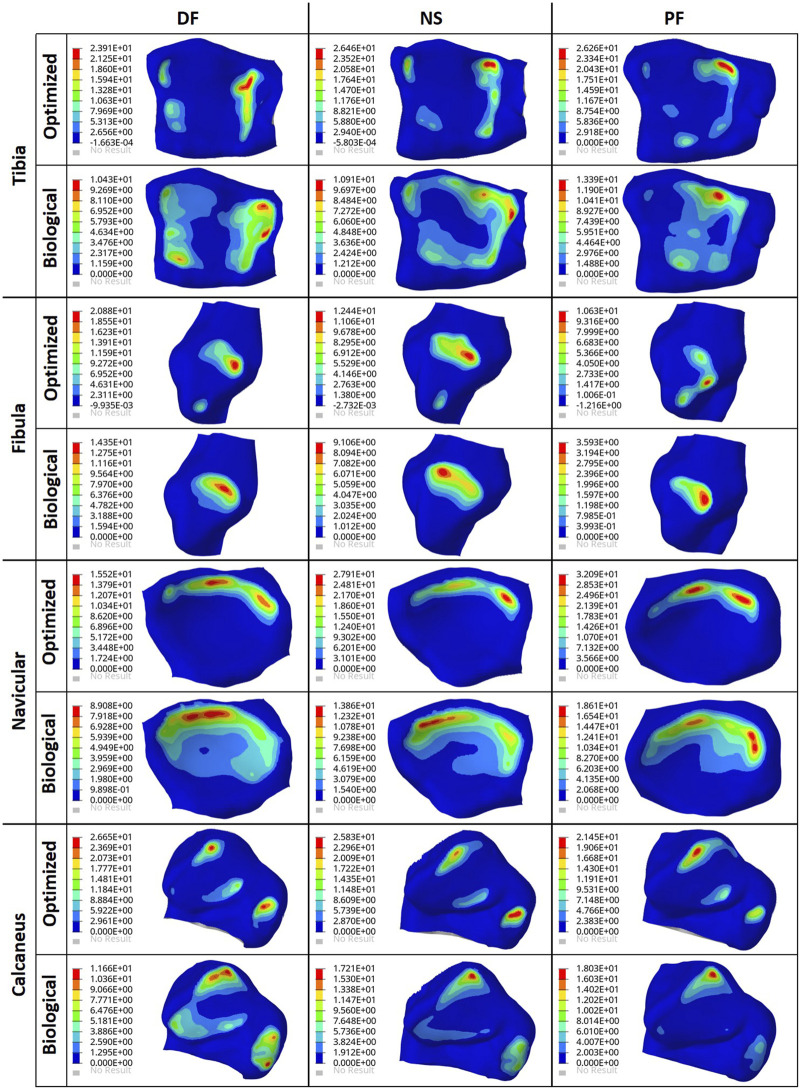
Contact pressure (MPa) contours for the optimized implant and biological talus.

The effect of the optimized implant on the adjacent cartilages’ contact characteristics was considered. Since a higher pressure, beyond a certain limit, could lead to bone fracture (Liu et al., 2022a), any talus implant should ideally have contact pressures and areas similar to those of the biological bone. For comparison of the optimized and solid implants as well as the biological talus, the maximum contact pressures are plotted in Figure 8. For the optimized implant and biological talus, the contact pressure contours of the adjacent cartilages in DF, NS, and PF are shown in Figure 9. As for the solid implant, the equivalent contours are not shown since the contact pressure patterns were identical to those produced by the optimized implant, and exhibit negligible maximum pressure differences, as seen in Figure 8.

## 4 Discussion

### 4.1 Results discussion

Based on Figures 4, 5, the material appears to be distributed in a way that is mostly governed by the load transfer paths, and is removed in the sections that are less stressed. This leads to an optimal material distribution that reduces the implant’s mass while rendering it safe for implantation by reinforcing the sections that transfer the load. For the summarized masses in Figure 6A, it is evident that the optimized implant weighs considerably less than the solid, non-optimized one. This lightweight implant design is expected to provide the patient with a more natural feel similar to that offered by the biological bone.

As for the stresses in all three postures in the optimized implant, based on Figure 6B; Figure 7, they are lower than the defined 75 MPa stress constraint. Additionally, the overall SF (lowest of the three postures, in DF) under static loading conditions is approximately 15.7. Accordingly, the optimized implant is deemed to be safe given its high SF. Additionally, as expected, it has higher stresses than the solid implant due to the availability of less material. For the biological talus, given its low elastic modulus, it experienced the highest stress across all three postures.

Finally, based on Figures 8, 9, the optimized and solid implants’ contact pressures on the adjacent cartilages are identical in all three postures. While this could partially be attributed to the nature of the material used for the implant which is considerably stiffer than cortical bone (Liu et al., 2022a), it is more likely that this is due to the unchanged articular surfaces (since they are a part of the non-design space). The biological bone has the lowest contact pressures with a significant peak pressure difference, relative to the implants. It produced peak pressures approximately 15.9%–66.2% lower than those produced by both implants. Additionally, on average, the contact areas appear to be higher for the biological talus in comparison to the implants. This could be attributed to the less stiff nature of cortical bone, but more significantly, this is likely due to the cartilage layer on the biological talus. Hence, adding an artificial cartilage layer on the implant surface could help in alleviating contact pressure peaks and guard against bone fractures near the bone/implant interfaces. Increased contact areas and reduced contact pressures due to the addition of a compliant layer on top of the metal-based implant were also found in a recent study (Liu et al., 2022b).

The results of this study deem the usage of optimized universal implants a feasible alternative to traditional custom-made ones. The patient is expected to benefit from a more natural feel as a result of the optimized implant’s lightweight nature as well as reduced times until surgical implantation given the universal nature. More generally, this study can help define a framework on how to approach the optimization of talus implants to obtain higher-performance and more economical total talus replacements.

### 4.2 Limitations and future work

Future work on the topic should focus on addressing the limitations of the present study. The optimization should be conducted for different types of loading such as dynamic (based on the human gait cycle), fatigue, and impact loading, that is not only limited to a single plane (sagittal in this research), but also to coupled out of plane loading and motion such as in the frontal plane. For the adjacent bones, more subjects could be used for optimization based on a variety of higher-quality bone geometries, rather than a single geometry. Moreover, more anatomically accurate ankle joint setups can be used in which both cortical and cancellous bone properties are considered, alongside the use of ligaments and muscles. Ligaments were not included in this comparative study given that the same postures and boundary conditions were used for all three tali and static loading was assumed. While the ligaments can play a significant role under dynamic loading, ligament usage is expected to have a minimal impact on the results when loads are applied statically (Liu et al., 2022a). To possibly reduce contact pressures, the usage of other materials (or a combination) could be explored. Additionally, to reduce the potential of stress concentrations without significantly increasing the implant’s weight, the use of a porous structure within the design space can be explored. This would likely offer enhanced energy absorption and buffering effects, which are beneficial for the long-term performance of the implant (Peng et al., 2022).

Finally, it is worth noting that, while a constant elastic modulus was assumed throughout the implant, in practice, the mechanical behavior of the component may vary spatially. In particular, lower thickness sections realized through AM have been shown to possess a lower elastic modulus as compared to their bulk equivalent (Danielli et al., 2023). Hence, the computed geometry may not exhibit the intended optimality and safety criteria in the additively manufactured part. Consequently, the fabricated implants would need to be mechanically tested to validate the study’s results and ensure safety.

## 5 Conclusion

This study focused on optimizing a universal talus implant, for total talus replacement, using topology optimization. For three postures, an FE model was developed for the biological talus, the solid implant, and similarly for the optimized implant post-optimization.

The major findings pertaining to the optimized universal implant are as follows. Its mass is significantly reduced (by approximately 66.6%). Based on maximum von Mises stresses in all three postures, it evidently satisfies the stress constraints (≤75 MPa) set in the optimization. Additionally, it is deemed to be safe in that it withstands 2000 N of static loading in all three postures with a safety factor of 15.7. This is based on the worst-case posture, dorsiflexion, with the highest maximum stress among all three. As for its effect on the surrounding cartilage, its maximum contact pressures were identical to those of the solid implant, therefore that aspect remained unaffected by the optimization.

## Data Availability

The raw data supporting the conclusion of this article will be made available by the authors, without undue reservation.
